# Double Trouble: A Case Report on the Surgical Management of Dual Intracranial Metastases

**DOI:** 10.7759/cureus.59582

**Published:** 2024-05-03

**Authors:** Ilko Ilyov, Stefan Burev, Asen Hadzhiyanev, Daniel Kolev, Stela Petrova, Petar-Preslav Petrov, Kiril Ivanov, Plamen Penchev

**Affiliations:** 1 Faculty of Medicine, Medical University of Plovdiv, Plovdiv, BGR; 2 Department of Neurological Surgery, University Hospital "St. Ivan Rilski", Sofia, BGR; 3 Department of General and Clinical Pathology, University Multi-profile Hospital for Active Treatment and Emergency Medicine (UMHATEM) Pirogov, Sofia, BGR; 4 Department of Anatomy, Histology and Embriology, Medical University of Plovdiv, Plovdiv, BGR

**Keywords:** neurosurgery technique, neuronavigation, primary breast malignancy, cerebral metastasis, mri images

## Abstract

Intracranial metastasis disease (IMD) has proven to be a frequent secondary occurrence, usually for primary cancers such as lung, breast, and melanoma, which have a high possibility of metastasizing to the brain. Due to the reasons listed above, treatment and early diagnosis are incredibly challenging. In the past decade, medicine has developed much better imaging solutions and radiological and surgical approaches, increasing the postoperative survival prognosis and achieving more time-efficient results. It is still exceptionally difficult to be able to prevent what type of metastasis a patient might develop other than by using the tumor type or subtype. We present a case of a 51-year-old female patient entering the Neurosurgical Clinic at the University Hospital “St. Ivan Rilski” for operative treatment of a second metastatic lesion located on the left parietal lobe in January 2024. She had previously had an operative resection of an initial lesion located on the left temporal lobe in December 2023. Her medical history began in 2015 when her first diagnosis was a breast carcinoma, followed by operative treatment and radio-, chemo-, and targeted therapy. In 2020, due to metastases located in the bones, she had to undergo another treatment with chemotherapy as well as have a total hysterectomy done as a result of another metastasis. The patient did not provide any family history, nor did she confirm any past or current allergies to foods, drugs, etc. Under general inhalation anesthesia, the patient was placed in a park bench position to the right and had a Mayfield head holder applied. Through a left parietal craniotomy and neuronavigation, a tumor formation was revealed with the characteristic of a secondary lesion. A gross total resection was achieved through a microsurgical technique. Postoperatively, there were no further complications observed in the patient, and she was discharged on day five from the hospital with relief of her symptoms.

## Introduction

Brain metastases (BMs) are seen in almost one-third of adults with solid tumor malignancies and have proven to have devastating consequences regarding neurologic symptomatology and psychological impact, which can in turn lead to a change in the treatment plan as well as approach. In the United States, the incidence of BMs has increased in the past decade as a result of the advancement of imaging techniques and systemic treatment regimens. Incidence rates reach as high as 70-400,000 new cases every year [1,2,3]. Worldwide, intracranial metastasis disease (IMD) was discovered in 25,478 of 601,678 (4.2%) individuals who had been diagnosed with primary malignancy. The disease sites ranged in the median period from initial cancer diagnosis to IMD (5.2 (0.7, 15.4) months; for example, the lung site took 2.1 months, the kidney site 7.3 months, and the breast site 22.8 months) [4]. The surgical approach when it comes down to treatment plays a crucial role, and the resection of such pathologies results in immediate relief of intracranial hypertension as well as focal neurological deficits. Surgery also allows for a definitive histological diagnosis, which MRI or radiation therapy doesn’t provide [5,6].

Systemic medication therapy has been somewhat successful in managing brain metastases until recently. The limited efficacy of medications is commonly attributed to their lack of permeability via the blood-brain barrier (BBB).

Even the drug temozolomide, which is the norm for treating glioblastoma patients, has only modestly improved the prognosis for brain metastases. For brain metastases, temozolomide's total intracranial response rate as a single treatment was less than 10% for a variety of primary tumor types. Similarly, chemotherapies given for the primary tumor demonstrate very little intracranial efficacy [7].

The reasons listed above show the impact that BMs have on patient recovery. This case report aims to highlight the importance of surgery in treating intracranial pressure and neurological impairments brought on by brain metastases. In addition to offering instant relief, surgical resection makes it possible to obtain a conclusive histology diagnosis, which is crucial for informing subsequent therapy choices.

## Case presentation

We present a case of a 51-year-old female patient entering the Neurosurgical Clinic at the University Hospital “St. Ivan Rilski” for operative treatment of a second metastatic lesion located on the left parietal lobe in January 2024. She had previously had an operative resection of an initial lesion located on the left temporal lobe in December 2023. The patient presented with a clinical manifestation of a headache and seizures, which have been going on for two months. Her medical history began in 2015 when her first diagnosis was ductal carcinoma of the left breast, followed by a total mastectomy combined with radiotherapy, chemotherapy, and targeted therapy. In 2020, due to metastases located in the bones, she had to undergo another treatment with chemotherapy as well as have a total hysterectomy done as a result of another metastasis. The patient did not provide any family history, nor did she confirm any past or current allergies to foods, drugs, etc.

**Figure 1:**
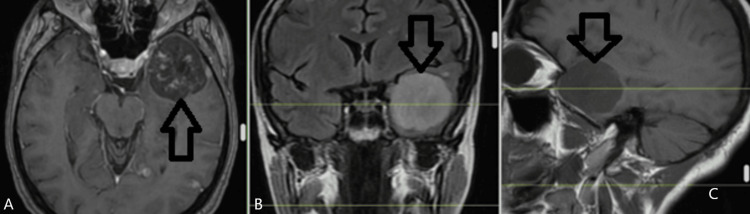
MRI shows a left temporal lobe lesion A: axial plane; B: coronal plane; C: sagittal plane

Upon physical examination, the patient had an overall good status. She was afebrile, and her skin and mucous membranes were presenting with a pale pink color. Her abdomen was soft on palpation. Upon percussion and auscultation, her respiratory system was intact, air exchange was not obscured, and there were no abnormal sounds monitored. Her cardiovascular health is also considered to be in excellent condition. Mobility in the extremities was fully intact, as was the fact that there was no swelling noted. The Glasgow Coma Scale (GCS) was 15, with no lateralizing deficit, no cranial nerve damage, and normal coordination. MRI imaging showed a left temporal lobe lesion (Figure 1). 

**Figure 2:**
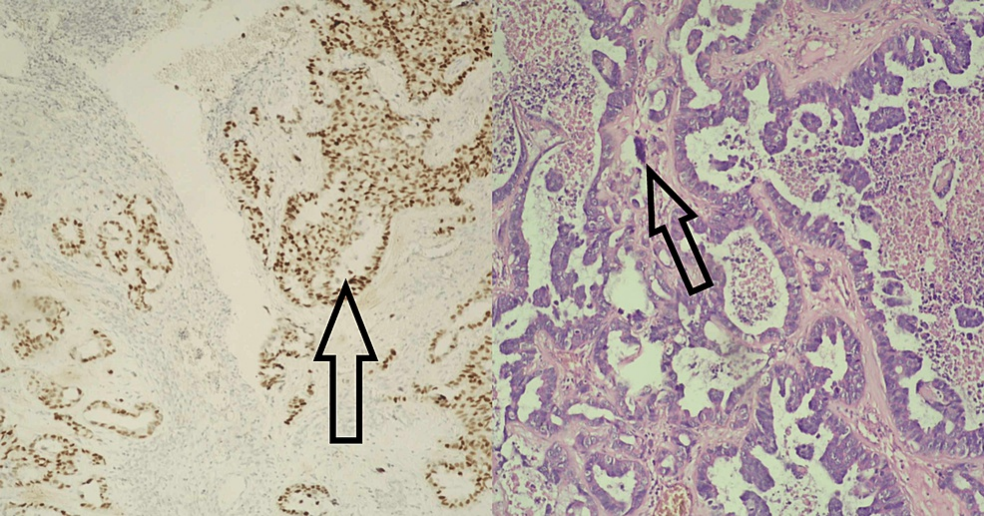
Immunohistochemical study performed Neoplastic cells express E-cadherin; HER-2 (3+); estrogen: strong positive nuclear reaction in more than 67% of tumor cells; progesterone: moderate positive nuclear reaction in more than 33-67% of tumor cells. HER-2: human epidermal growth factor receptor 2

Under general anesthesia, the patient was placed in a park bench position to the right and had a Mayfield head holder applied. Through a left frontotemporal craniotomy, an incision of the dura was made, and access through the superior temporal sulcus was ensured. Through neuronavigation and a microsurgical technique, a tumor formation was revealed with the characteristic of a secondary lesion. A gross total resection was achieved. Pathology confirmed that the lesion had metastasized from breast carcinoma. Immunohistochemical evaluation of cerebral parenchyma yielded infiltration of low-differentiated (G3) ductal adenocarcinoma of breast origin with micropapillary areas and extracellular mucinous production (Figure 2).

**Figure 3:**
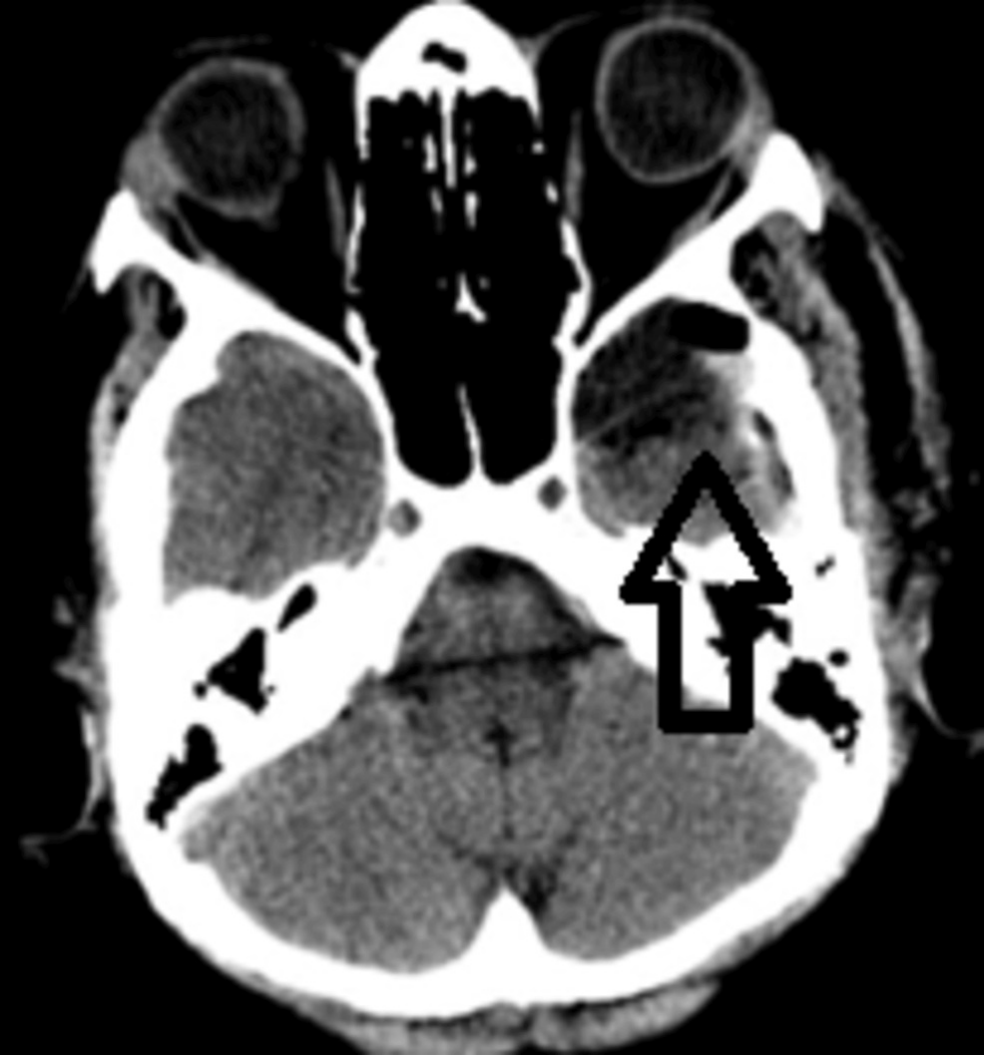
An MRI shows total extirpation of the malignant mass MRI: axial plane

Postoperative MRI showed no ischemic or hemorrhagic events (Figure 3).

Postoperatively, the patient was mobilized a day after the intervention. Surgery-related complications were not observed. The patient had an improved neurological status and was discharged on day five. Chemotherapy and radiotherapy treatment followed, along with regular MRI check-ups. Unfortunately, one month after the removal of the temporal metastasis (December 2023), a parietal one was imaged.

**Figure 4:**
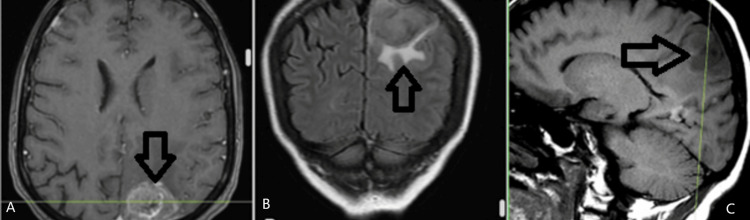
Left parietal lobe lesion imaged through MRI A: axial plane; B: coronal plane; C: sagittal plane

The patient was admitted to the Neurosurgical Clinic at the University Hospital “St. Ivan Rilski” with recurring headaches and dizziness that have been going on for seven days. MRI discovered a tumor formation located at the left parietal lobe suspicious of a metastasis (Figure 4).

**Figure 5:**
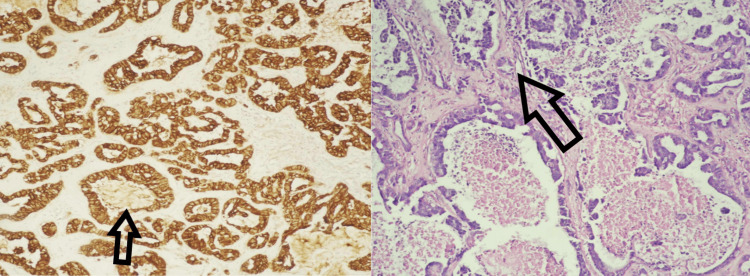
Immunohistochemical study performed Neoplastic cells express E-cadherin; HER-2 (3+); estrogen: strong positive nuclear reaction in more than 67% of tumor cells; progesterone: moderate positive nuclear reaction in more than 33-67% of tumor cells. HER-2: human epidermal growth factor receptor 2

Under general anesthesia, the patient was placed in a park bench position to the right and had a Mayfield head holder applied. A “Lazy S” type of incision was performed and through a left parietal craniotomy and neuronavigation combined with a microsurgical technique, a tumor formation was revealed and was successfully extirpated. The pathology results confirmed that it was a recurrent metastasis from breast cancer because there were nests of polymorphic cells with an avid characteristic of ductal carcinoma of the breast (Figure 5).

**Figure 6:**
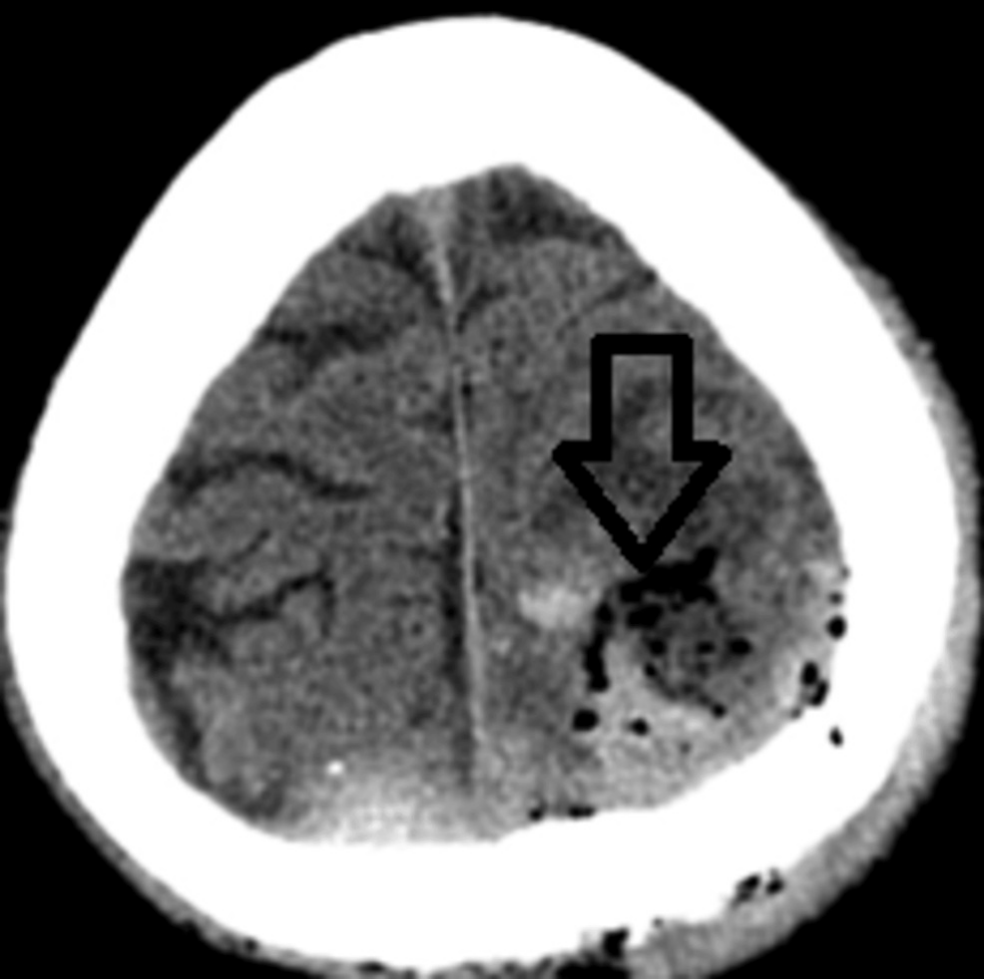
An MRI shows evidence of total extirpation of the tumor mass MRI: axial plane

The postoperative MRI showed a total extirpation of the tumor mass (Figure 6).

Postoperatively, the patient was verticalized on the day after the intervention. We did not observe any complications from the surgery. The patient was discharged on day six with relief from her symptoms. She is now undergoing chemo and radiotherapeutic treatment and is expected to have follow-up imaging.

## Discussion

Brain metastasis still remains one of the most common pathologies in the United States, accounting for the fact that 8-10% of patients with cancer will develop brain metastasis, which in turn leads to 200,000 new patients every year. Brain metastases expand through the creation of new blood vessels or the appropriation of preexisting ones; the brain microenvironment fosters tumor growth, tumor cell survival, and therapy resistance [8]. In the case of metastatic melanoma and lung adenocarcinoma, for instance, the incidence proportion of patients with brain metastases at diagnosis is estimated to be over 25%; for metastatic renal cell cancer, it is 10%; for metastatic breast cancer, it is 7%; for metastatic head and neck cancer, it is 5%; and for nonesophageal metastatic gastrointestinal cancers, it is 2% [9]. Moreover, most patients will develop brain metastases after the first diagnosis [10]. These facts correspond to our case and showcase the aggressive nature of breast carcinoma.

The advancement of diagnostic and therapeutic approaches over the years has contributed to a great deal of improvement. Early ventures at establishing guidelines were vague and focused on the implementation of palliative care, steroids, and whole brain radiation therapy (WBRT), with the understanding that the application of chemotherapy and surgery was not supported by any controlled, randomized studies [11]. The initial attempts to develop guidelines through a more objective and structured process did not yield meaningful guidance due to insufficient evidence of high enough quality to make firm recommendations [12]. In 2019, the American Society for Radiation Oncology (ASTRO), the Society for Neuro-Oncology (SNO), and the American Society of Clinical Oncology (ASCO) agreed that a single document addressing the treatment of brain metastases from nonhematologic solid tumors was necessary. Therefore, a conclusion was drawn that patients with intracranial metastases benefit from surgical intervention and are more likely to benefit if they have large tumors with mass effects as opposed to ones with multiple metastases or uncontrolled pathology. For melanoma, breast cancer, and non-small-cell lung cancer, many regimens were suggested. Patients with one to four unresectable brain metastases, excluding small-cell lung carcinoma, should be administered stereotactic radiosurgery (SRS) alone if they are asymptomatic and have no other choices for systemic therapy. Patients with one to two resected brain metastases should be given SRS alone in the surgical cavity. For some patients, SRS, whole-brain radiation therapy, or a combination of both might be appropriate alternatives [13]. Our patient is currently being treated with chemo and radiotherapy and is awaiting a follow-up examination. The current treatment corresponds to the guidelines administered by ASTRO.

Alessandra Fabi et al. point out that breast cancer was the tumor that had the longest time to recur in the brain (46 months), most likely due to the benefits of early detection and the availability of efficient therapies. Alessandra Fabi et al. also point out that in 31% of instances, a local therapy strategy combining stereotactic radiosurgery and surgery was used. Notably, there was a substantial difference in survival at two years between the regional/systemic method (whole brain radiation and chemotherapy) and the local technique, which demonstrated higher survival outcomes [14]. This aligns with the surgical approach chosen in our case.

The mechanism by which brain metastases occur is intricate and involves the initial breast cancer cells invading surrounding tissue and arteries, moving through the circulatory system, colonizing and growing in the brain parenchyma, and all of these things. On average, the treatment process for breast cancer takes 32 months after the first cancer diagnosis. This indicates that in contrast to other cancers, breast cancer tumor cells require a longer time to establish the capacity to cross the blood-brain barrier (BBB) and invade the brain [15,16]. José Pablo Leone et al. point out that, depending on the tumor subtype, individuals with progressive systemic illness at the time of brain metastasis formation should have their systemic medication modified [17]. This reflects our case due to the fact that we had two brain metastases that occurred in two consecutive years. Our patient previously had uterine and bone metastases before the intracranial ones occurred.

Immunohistochemical markers such as human epidermal growth factor receptor 2 (HER-2), E-cadherin, estrogen, and progesterone are associated with brain metastases and pose a greater risk in patients with breast cancer. Our case does depict such markers and is in correlation with recent literature [18].

## Conclusions

This case report highlights the critical importance of surgical intervention in the treatment of intracranial metastatic disease (IMD). Our patient's intracranial pressure and focal neurological impairments were promptly relieved by careful surgical resection, which facilitated a speedy recovery and symptom-free release.

The favorable result emphasizes the significant benefit of surgery in reducing the catastrophic effects of brain metastases, both in terms of neurological function and in terms of delivering a conclusive histological diagnosis that is essential for informing subsequent treatment choices. With further developments in imaging methods, systemic treatments, and surgical strategies, there is still hope for better patient outcomes and a higher standard of living for those with IMD. 
